# The prevalence of naturally acquired swimming ability among children in Bangladesh: a cross sectional survey

**DOI:** 10.1186/1471-2458-14-404

**Published:** 2014-04-27

**Authors:** Aminur Rahman, Michael Linnan, Saidur Rahman Mashreky, Mohammad Jahangir Hossain, Fazlur Rahman

**Affiliations:** 1 International Drowning Research Centre–Bangladesh (IDRC-B), House–B 162, Road–23, New DOHS, 1206 Dhaka, Mohakhali, Bangladesh; 2 The Alliance for Safe Children (TASC), Atlanta, USA; 3 Centre for Injury Prevention and Research, Bangladesh (CIPRB), Dhaka, Bangladesh

**Keywords:** Swimming ability, Naturally acquired swimming ability, NASA, Child drowning, Drowning prevention, Bangladesh

## Abstract

**Background:**

Most rural homes in Bangladesh have ponds nearby to serve as household water sources. As a result children of all ages are exposed to water bodies on a daily basis. Children learn to swim early in childhood from peers and relatives in a natural process that involves play and structured learning. In a large, national injury survey in Bangladesh, the ability to swim was associated with reduced risk of drowning. This study determines the prevalence of swimming ability in children in Bangladesh as a step in assessing whether this is a potential component of a national drowning prevention program.

**Methods:**

A descriptive study design using a subset of a national sample survey determined the prevalence of naturally acquired swimming ability (NASA) reported by children of rural and urban communities in Bangladesh. A total of 2,598 households (1,999 rural and 599 urban) housing 4,336 children (2,263 male and 2,073 female) aged 5-17 years were chosen from 4 randomly selected districts using multistage random sampling. NASA was defined as the ability to cross 25 meters of water deeper than the child’s height using any body movement for self-propulsion.

**Results:**

Reported NASA was greater in males (55.6%) than females (47.9%) and among rural children (57.8%) than urban children (25.5%) for children 5-17 years. The proportion reporting NASA increased with increasing age. At age 5, 5.8% of males and 6.3% of females reported NASA, rising to 84.3% of males and 70.7% of females by age 17. By age 17, 83.1% of rural children and 57.5% of urban children reported NASA.

**Conclusion:**

Most children in Bangladesh report being able to swim 25 meters and learning it by middle childhood. Reported NASA is higher for males than females and for rural children than urban children. High rates of swimming appear to be achievable in the absence of pools and a swim-teaching industry. This may facilitate development of a low cost, national drowning prevention program with swimming an integral part.

## Background

Bangladesh is largely a riverine delta formed in part by the Padma, Jamuna, and Meghna Rivers flowing through it and emptying into the Bay of Bengal. Villages are usually surrounded and intersected by rivers and canals. Rural homes lack piped water supplies and almost all homes have ponds in close proximity which serve as water sources for household water, bathing, cooking and often raising fish and providing water for animals. As a result, exposure to water bodies is very high in daily life in rural Bangladesh. Children use natural water bodies for bathing and playing and this high level of water exposure contributes to the high rate of child drowning in Bangladesh [1]. The Bangladesh Health and Injury Survey (BHIS) done in 2003 showed drowning was a leading killer of children after infancy and through age 17. BHIS found that more than 18,000 children aged 1-17 years drowned in 2003, the year of the survey [2]. The fatal drowning rate was 28.6 per 100,000 children 1-17 and 32.4 and 16.7 per 100,000 males and females 1-17 years respectively. Other child injury research in Bangladesh has found drowning a leading cause of child death [3]. Studies in other Asian countries also have shown high rates of drowning, examples being Thailand (37.9 per 100,000 1-17 yrs) and Cambodia (37.6 per 100,000 1-17 yrs) [4,5].

While placing children at risk of drowning, the daily exposure to water also offers children the opportunity to learn to swim, usually taught informally by their peers and relatives. A nested case-control study in BHIS found an association between increased risk of drowning and lack of swimming ability (OR = 4.5; 95% CI 1.25-19.4) [2]. BHIS was followed by an anthropologic study on how natural swimming ability was acquired. The study depicted an informal but relatively structured process with water-play beginning very early in childhood and progressing in complexity until children had learned to swim by middle childhood [6]. Both sexes participated and ponds were the most frequent places of teaching. The study also showed that the process of learning was potentially hazardous. Study respondents described fatal drowning occurring in children learning to swim. The association of swimming with reduced drowning risk and the demonstration of swim learning as a cultural norm raised the possibility of swimming being used as a drowning intervention if it could be made safer. The objective of this study was to examine a subset of the BHIS cohort in further detail to determine the prevalence of swimming ability among children in Bangladesh.

## Methods

The Survey on Swimming Skills, Household Injury Hazards and Children Sleep Patterns was a cross-sectional survey done using a subset of the BHIS survey immediately following it in 2003. A total of 2,598 households (1,999 rural and 599 urban) housing 4,336 children (2,263 male and 2,073 female) aged 5-17 years were chosen from 4 randomly selected districts using multistage random sampling. Urban households were chosen from metropolitan Dhaka and mohallas (the administrative seats of non-metropolitan districts) from the other 3 districts. Rural households were chosen from unions in upazilas (lowest administrative area of non-metropolitan districts). Trained interviewers visited the homes selected in each area and completed interviews. There were no refusals to participate in the interviewers.

A questionnaire was used to collect information from caretakers and children. It was tested and retested for clarity, reliability, ease of use and data entry efficiency with serial testing among multiple groups of urban and rural residents. In the swimming skills module, naturally acquired swimming ability (NASA) was defined as a child’s ability to cross 25 meters of water deeper than the child’s height using any body movement for self-propulsion. It was determined by a trained interviewer asking the following question in Bangla: ‘If (name) fell into a pond that was 25 meters across and deeper than (name’s) height could (name) move across the water to the other side and climb out?” The interviewer administered the questionnaire to the caretaker of all children aged 5-10 years old (n = 2,255) and with the children themselves if 11-17years old (n = 2,081). Each respondent was asked about their swimming ability, the age at which they learnt to swim, who taught them to swim, and the type of water body in which they learnt to swim.

Data was entered with Epi Info software and results were analyzed using SPSS software. A weighted analysis was done using the rural-urban weights for the combined sample and an un-weighted analysis was done for the rural and urban areas separately. The chi-square test for proportions was used to compare categorical responses.

Ethical clearance was obtained from the Ethical Review Committee of the Institute of Child and Mother Health, Dhaka, Bangladesh. Oral consent was obtained from the respondents prior to conducting the interviews. Parents gave informed consent for all children regardless of age.

## Results

**Figure 1:**
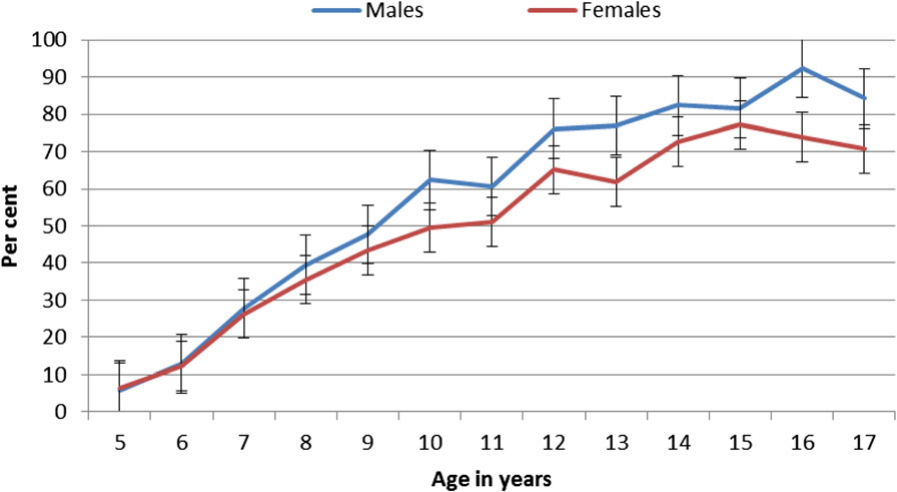
Reported naturally acquired swimming ability according to age and sex.

The proportion of children reporting NASA (as per our definition of being able to cross 25 meters of water deeper the child’s height using any body movement for propulsion) among those 5-17 years old was 51.9% (n = 2,252). Higher levels of NASA were reported among males (55.6%, n = 1,258; 95% CI 53.5-57.6%) than females (47.9%, n = 994; 95% CI 45.7-50.1%). The proportion of children reporting NASA increased with increasing age. At age 5, 8% (n = 10) of males and 6.3% (n = 10) of females reported NASA, rising to 84.3% (n = 91) of males and 70.7% (n = 65) of females by age 17 (Figure 1).

**Figure 2:**
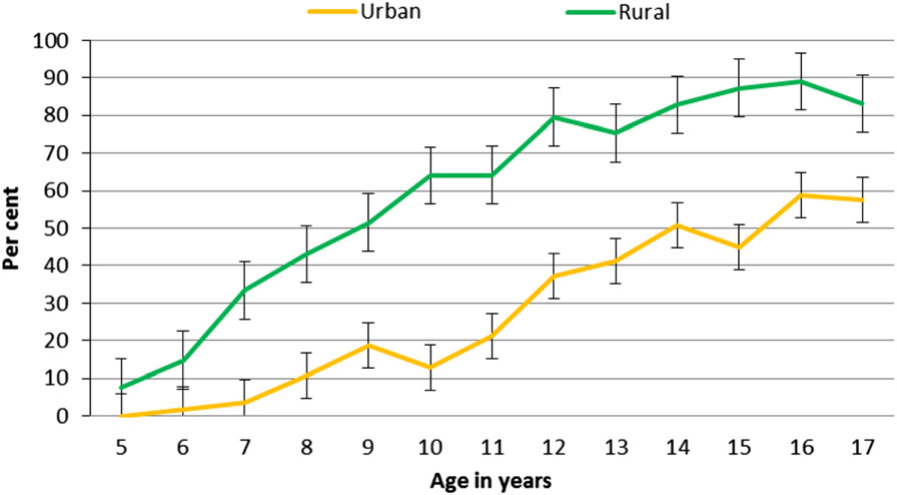
Reported naturally acquired swimming ability according to age and residence area.

Among children 5-17 years of age, the proportion reporting NASA was significantly higher (p < 0.001) in rural children (57.8%, n = 2,051; 95% CI 56.1-59.4) than urban children (25.5%, n = 201; 95% CI 22.5-28.7). By age 17 years, the proportion of rural children reporting NASA ( 83.1%, n = 133; 95% CI 76.2-88.4) was significantly higher (p < 0.001) than urban children (57.5%, n = 23; 95% CI 40.9-72.9) (Figure 2).

The most common place of learning swimming was ponds (72.1%). A small proportion of children reported that they learnt in rivers and lakes, 15.5 and 11.7% respectively. Urban-rural differences can be seen with one urban child learning in a pool and only rural children learning in lakes (Table 1).

**Table 1:** Place of learning to swim in urban and rural areas

**Place**	**Urban**	**Rural**	**P value for difference**
	**n (%)**	**n (%)**	
Pond	175 (7.5)	1148 (70.6)	<0.001
River	21 (10.5)	329 (16.0)	<0.001
Pool	1 (0.5)	0 (0.0)	UTD*
Lake and other places	0 (0.0)	263 (12.8)	UTD*

*Unable to determine due to one proportion being zero.

Regarding who taught the child, more than one third (40.3%) were taught by parents, and in rural settings parents taught children almost twice as often as in urban settings. Slightly more than a third (37.8%) learnt how to swim by themselves. About 9.5, 6.7 and 5.0% of the children received swimming teaching from friends/peers, siblings and other relatives, respectively (Table 2).

**Table 2:** Who taught child in urban and rural areas

**Teacher**	**Urban**	**Rural**	**P value for difference**
	**n (%)**	**n (%)**	
Parents	46 (22.9)	862 (42.0)	<0.001
Siblings	19 (9.5)	130 (6.4)	0.08
Other relatives	17 (8.5)	95 (4.6)	0.40
Friends/peers	26 (12.9)	189 (9.2)	<0.02
Self	90 (44.8)	762 (37.2)	<0.001

## Discussion

The study examined swimming ability that was acquired informally from parents, siblings, peers or was self-taught. For that reason, it was termed naturally acquired swimming ability (NASA) to distinguish it from swimming skill acquired from trained, certified instructors in formal programs as occurs in many high income countries (HICs). The distance of 25 meters provided a measurable distance standard for NASA. Telling a story provided a standardized way of presenting the operational definition of NASA to a parent or a child. The 25 meter distance was chosen for practical reasons. It represents the size of the average pond making it easy for the respondents to answer the swimming questions [7]. It also coincides with the length of many swimming pools used in commercial swim teaching, thus providing a measure of comparability.

Importantly, it sets a distance standard that has objective, epidemiological evidence showing that achieving this distance results in a statistically significant association with reduced drowning. The nested case-control study in the BHIS used the same definition and story-based method of ascertainment. That study (field work 2003) found children reporting NASA were 4.5 times less likely to die from drowning than children who did not [2]. Similar associations were seen using the same standard definition and story-based ascertainment methodology in nested case-control studies in surveys in Thailand (field work 2004) and Cambodia (field work 2006) [8].

Findings from the anthropological research show in early childhood there are no differences in male or female child participation in the water-play or other water-based activities that progress to NASA [6]. However in middle childhood, female physical development results in girls no longer participating in mixed gender water play or other in-water activities. This conservative cultural norm was validated in the anthropological research. These cultural factors mediate lower participation, thus females are less likely to report NASA than males as found in this study. A similar rate of self-reported NASA from ages 4 through 7 is evident in Figure 1, whereupon the curves diverge and become clearly different by age 10. However, in spite of this, a large majority of females (70.7%) reported achieving NASA by the end of childhood.

Rural-urban differences can be explained by the abundance of open water bodies in rural areas and the relative absence of them in the urban setting. Notably, the anthropological study found that some urban children actually learned swimming in rural settings for two reasons: 1) Many urban residents are recent migrants whose children initially learnt to swim in rural settings; and 2) .Many families return to their rural villages during annual holidays and young children acquire NASA through joint play with rural peers [6].

The anthropological research on the process of acquiring natural swimming ability showed it is an integral part of rural Bangladeshi culture. The high proportion of children that report NASA documented in this survey attests to the effectiveness of this practice as a pervasive cultural norm. However, the anthropological research as well as many common anecdotes indicates there are hazards associated with the practice. This level of risk will not be acceptable in a formal program.

Further work is underway to understand how to safely develop NASA as early in childhood as possible, as soon as a child is developmentally capable of learning. BHIS showed the median age of drowning in Bangladesh was between 2 and 3 years of age. Fatal drowning rates are highest in early childhood and drop rapidly after 5 years of age. Thus, to be most effective a drowning prevention program needs to begin in early childhood. The longitudinal SwimSafe effectiveness study showed children 4 years old were capable of safely learning basic swimming [9]. A small number of 3 year old children also participated, but were selected on the basis of being at or above growth and development norms for children in Bangladesh. Thus current evidence does not yet answer the question of how early in childhood Bangladeshi children can safely begin to learn to swim.

The demonstration of high levels of NASA achieved through use of existing bodies of water is an important point. Swim training infrastructure used in high income countries primarily is composed of in-ground pools, trained life guards and certified swimming instructors. These are not economically possible, nor are they practically or culturally appropriate at present in a country such as Bangladesh. Low costs and cultural acceptability will be key requisites for developing a national drowning prevention program where swimming is an integral part. If the work currently underway is able to develop a safe basic swimming and safe-rescue training protocol using the same natural water bodies children currently use to attain NASA, it may permit basic swimming to be part of a national drowning prevention program.

There are limitations in the study. A major one is that ability to swim was reported either by parents of young children or self-reported by older children and were not validated by test. Studies have shown varying results on whether self-reported data on swimming ability correctly estimates actual performance when tested [10]. This study used reports that were not tested. While a potential limitation, the association of reduced drowning risk seen in the case-control study in BHIS and the similar studies in other countries were also based on un-validated reports of the same definition obtained in the same standard manner. Whether it is a true estimate of ability to swim 25 meters or only the belief of being able to swim 25 meters, reporting it was associated with actual measured reduction in drowning risk in the case-control studies.

Another limitation is the lower age limit for inclusion. The lower bound of 5 years was used in the BHIS national survey and this study used a subset of the national sample. Therefore children 3 and 4 years old are not included. The anthropological study on swimming skills showed swim learning begins early in the second year of life in rural villages and some children are able to swim 25 meters at 3 years of life. Evidence currently available from the SwimSafe cohort study does support healthy 4 year old children being included [9]. However, it does not yet establish whether this is the case for children 3 years old or for older children with delayed growth and development. Currently research is in progress to determine this.

## Conclusions

Most children in Bangladesh begin informal, natural swim learning in early childhood and report acquiring NASA by middle childhood. Reported NASA is higher for males than females and for rural than urban children. High rates are achievable in the absence of pools and a formal swim-teaching industry. This may facilitate development of a low cost, national drowning prevention program with swimming an integral part if safety issues can be properly addressed. The ability to use existing bodies of water avoids a major constraint of having to create a swim teaching infrastructure that is currently impossible in many developing countries.

## Competing interests

The authors declare that they have no competing interests.

## Authors’ contributions

AR, FR and ML conceived the study, participated in the design, implementation, and analysis. AR wrote the first draft of the paper and FR and ML contributed to the manuscript. SRM was involved in instruments development, supervised field work, participated in analysis of data, and contributed to the manuscript. MJH contributed to the manuscript. ML and FR are guarantors. All authors read and approved the final manuscript.

## Authors’ information

The authors have been involved in research activities on the epidemiology of childhood drowning and its prevention for the last decade in Bangladesh.

## Pre-publication history

The pre-publication history for this paper can be accessed here:

http://www.biomedcentral.com/1471-2458/14/404/prepub
